# Systematic mapping of TF-mediated cell fate changes by a pooled induction coupled with scRNA-seq and multi-omics approaches

**DOI:** 10.1101/gr.277926.123

**Published:** 2024-03

**Authors:** Muyoung Lee, Qingqing Guo, Mijeong Kim, Joonhyuk Choi, Alia Segura, Alper Genceroglu, Lucy LeBlanc, Nereida Ramirez, Yu Jin Jang, Yeejin Jang, Bum-Kyu Lee, Edward M. Marcotte, Jonghwan Kim

**Affiliations:** 1 Department of Molecular Biosciences, The University of Texas at Austin, Austin, Texas 78712, USA;; 2 Department of Biomedical Sciences, Cancer Research Center, University at Albany, State University of New York, Rensselaer, New York 12144, USA

## Abstract

Transcriptional regulation controls cellular functions through interactions between transcription factors (TFs) and their chromosomal targets. However, understanding the fate conversion potential of multiple TFs in an inducible manner remains limited. Here, we introduce iTF-seq as a method for identifying individual TFs that can alter cell fate toward specific lineages at a single-cell level. iTF-seq enables time course monitoring of transcriptome changes, and with biotinylated individual TFs, it provides a multi-omics approach to understanding the mechanisms behind TF-mediated cell fate changes. Our iTF-seq study in mouse embryonic stem cells identified multiple TFs that trigger rapid transcriptome changes indicative of differentiation within a day of induction. Moreover, cells expressing these potent TFs often show a slower cell cycle and increased cell death. Further analysis using bioChIP-seq revealed that GCM1 and OTX2 act as pioneer factors and activators by increasing gene accessibility and activating the expression of lineage specification genes during cell fate conversion. iTF-seq has utility in both mapping cell fate conversion and understanding cell fate conversion mechanisms.

Master transcription factors (TFs), which control cellular identity and development, can often change cell fate upon induction in a process known as reprogramming, best exemplified by somatic cell reprogramming in which overexpression of four TFs generates induced pluripotent stem (iPS) cells from terminally differentiated cells (Takahashi and Yamanaka 2006; Takahashi et al. 2007; Yu et al. 2007; Park et al. 2008). Similarly, induction of a single master TF is often sufficient to reprogram cells in certain contexts; for example, ectopic expression of *Myod1* in fibroblasts generates myoblasts, and *Gata1* overexpression converts avian myelomonocytic cells into eosinophils, thromboblasts, and erythrocytes (Choi et al. 1990; Kulessa et al. 1995). Additionally, ectopic expression of individual trophoblast TFs, such as *Cdx2*, *Gata3*, or *Arid3a*, has been shown to convert embryonic stem (ES) cells to trophoblast-like cells (Niwa et al. 2005; Ralston et al. 2010; Rhee et al. 2014; Blij et al. 2015).

Reprogramming has enormous potential for regenerative medicine, as it enables the generation of desired cell types. To systematically test the potential of individual TFs in converting cell fates, recent studies have performed large-scale overexpression screens (Parekh et al. 2018; Nakatake et al. 2020; Ng et al. 2021; Joung et al. 2023) in human pluripotent stem cells, which are plastic and capable of differentiating into all three germ lineages and even extraembryonic lineages in vitro (Keller 1995; Smith 2001; Cho et al. 2012; Toyooka 2021). Although these studies successfully identified numerous TFs triggering differentiation of pluripotent stem cells, one study relying on a reporter was unable to annotate lineage specification (Ng et al. 2021), and the other studies using a constitutive promoter for the overexpression were unable to control the duration of TF induction (Parekh et al. 2018; Joung et al. 2023). Furthermore, none of the studies investigated the dynamic changes in the transcriptome or the action mechanisms of potent TFs during cell fate conversion.

The advent of single-cell RNA sequencing (scRNA-seq) has revolutionized our understanding of tissue and organ behavior at the level of individual cells (Ofengeim et al. 2017; Hwang et al. 2018; Papalexi and Satija 2018; Svensson et al. 2018; Madissoon et al. 2020; Li et al. 2020; Paik et al. 2020; Shiau et al. 2021). With scRNA-seq, cellular heterogeneity within an organ can be profiled at an unprecedented resolution, enabling the unbiased investigation of the single-cell transcriptome in a massively parallel manner. This approach has been used to study cellular heterogeneity in normal and abnormal settings and has also been combined with other high-throughput techniques, such as RNA interference, CRISPR-Cas9-mediated perturbation, and overexpression screens, to address numerous questions in biology and medicine without relying on a limited number of reporter genes (Adamson et al. 2016; Dixit et al. 2016; Jaitin et al. 2016; Aarts et al. 2017; Datlinger et al. 2017).

Here, we introduce iTF-seq, a novel method for a pooled induction screen of individual TFs with cell fate conversion potential. In this method, ectopic expression of TFs is performed using a transposon-based doxycycline (Dox)-inducible system, which enables greater transfection and genomic integration efficiency for the generation of individual cell lines for TF induction. Our analysis pipeline detects the induced TF (iTF) in each cell and profiles the resulting transcriptome that emerges from this induction. iTF-seq also allows subsequent multi-omics approaches through metabolic biotinylation of TFs for protein–DNA interaction (PDI) and protein–protein interaction (PPI) mappings centered on the potent TFs. In a pilot test of 80 TFs, iTF-seq identified multiple novel TFs with rapid cell fate conversion potential toward specific lineages, which underscores the importance of TFs in cell fate determination and provides valuable insights into the generation of desired cell types by TFs. The approach will serve as a framework for understanding common or unique TF-mediated cell fate conversion mechanisms.

## Results

### Construction of an SBFB vector

**Figure 1.**
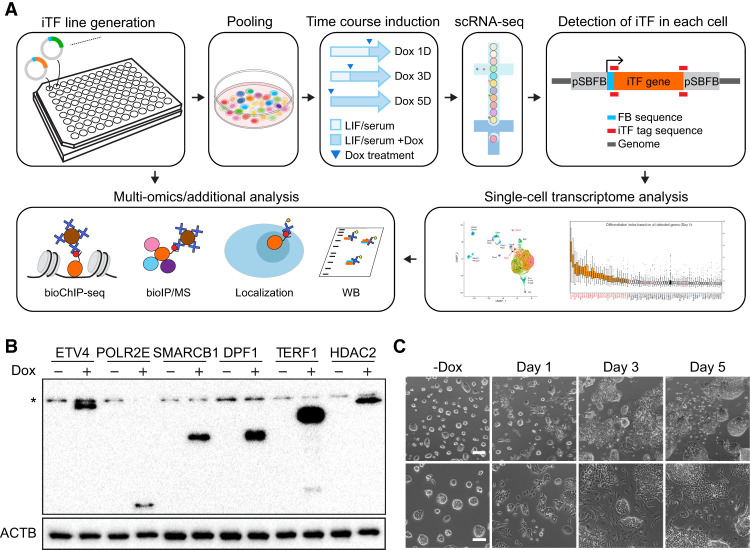
iTF-seq enables screening of numerous TFs with fate conversion potential. (*A*) Schematic representation of iTF-seq procedure and subsequent multi-omics approaches. (*B*) Representative western blot images showing inducible overexpression of biotinylated proteins in individual iTF lines detected by streptavidin-HRP. (*) Nonspecific bands, (Dox) doxycycline. (*C*) Morphology of 80 pooled iTF cell lines under uninduced condition (−Dox) and upon induction of TFs by treating Dox (0.5 μg/mL) for 1, 3, and 5 d. Scale bars, 100 μm or 200 μm (*upper*).

We developed a Sleeping Beauty (SB) transposon-based inducible vector in combination with a metabolic biotinylation (FB) system to enable the inducible expression of TFs in ES cells, which we refer to as the SBFB vector (Ivics et al. 1997; Kim et al. 2008; Hackett et al. 2010). Previous research has suggested that transposon-mediated approaches are superior to lentivirus methods for open reading frame (ORF) overexpression owing to the simplification of reagent preparation, reduced promoter silencing, and increased induction levels with higher genomic integration efficiency (Ng et al. 2021). In the SBFB system, the ectopically expressed proteins upon Dox treatment undergo biotinylation via a biotin ligase (BirA) that is constitutively expressed in J1 mouse ES cells (BirA-ES cells). This allows for streptavidin-mediated downstream multi-omics applications, including mapping of genomic targets and interacting partner proteins of the iTF of interest independently, in addition to the detection of protein induction and localization as previously described (Fig. 1A; Supplemental Fig. S1A,B; Kim et al. 2008, 2009, 2010).

### Generation of iTF lines

To investigate previously unreported TFs with cell fate conversion potential and to confirm the feasibility of the iTF-seq method, we generated cell lines for the induction of individual TFs in ES cells (iTF lines). A set of 80 TFs was selected as a proof of principle test. These TFs were chosen based on several factors, including cell or tissue type–specific expression patterns often displaying instructive roles in development (Zhang et al. 2019). Some of them are ubiquitously expressed, and we also included multiple previously reported TFs with reprogramming potential as a single factor, such as *Gata6* (Fujikura et al. 2002; Wamaitha et al. 2015), *Gata4* (Fujikura et al. 2002; Holtzinger et al. 2010), *Pdx1* (Bernardo et al. 2009), *Cited1* (Xu et al. 2018), *Cdx2* (Niwa et al. 2005; Blij et al. 2015), *Gli1* (Denham et al. 2010), *Arid3a* (Rhee et al. 2014), *Gata3* (Ralston et al. 2010), *Elf5* (Latos et al. 2015), *Nkx2-5* (Ruan et al. 2016), *Tfap2c* (Latos et al. 2015), *Fosl1* (Lee et al. 2018), and *Zfp36l1* (Tseng et al. 2017). Approximately 80% of the individual TFs tested did not have confirmed reprogramming potential from prior studies, as summarized in Supplemental Table S1. The cDNAs for the selected TFs were cloned into the SBFB vector, and 80 individual stable iTF lines were generated, as described in the Methods section, using transient expression of the SB transposase. This approach allowed us to generate stable iTF lines in a small-scale culture (96-well plate). We validated protein expression of all individual biotinylated TFs with streptavidin-HRP after 1 d of Dox induction (0.5 μg/mL) (Fig. 1B). For some iTF lines, we observed significant changes in morphology upon induction (Supplemental Fig. S2A).

### Detection of each cell with a specific TF induction by iTF-seq

To investigate transcriptome and cell fate changes caused by the ectopic induction of individual TFs at single-cell resolution, we performed scRNA-seq of a pool of 80 stable iTF lines and wild-type ES cells upon treatment with Dox for 1, 3, and 5 d. Similar to the test of individual iTF lines, a substantial proportion of cells within the pool showed a flattened morphology indicating differentiation upon 1 d of induction (Fig. 1C).

To detect a specific TF expressed ectopically in each cell, we applied a barcode-independent approach. Parts of the SBFB vector are transcribed with the TF coding sequences upon Dox induction, and we determined the junctional sequences between the vector and TF coding region (iTF tags) (Fig. 1A). This approach also distinguishes endogenous versus ectopic TFs within the cell, as iTF tags are unique for ectopic TFs and cannot be found in the endogenous transcripts. From the iTF-seq results, we collected all reads matched with the iTF tags and successfully detected individual cells expressing specific iTFs. Although we expected that each cell expresses only one iTF based on our experimental design (Fig. 1A), we observed cells expressing iTF tags associated with multiple iTFs. The results may arise from the cumulative errors from single-cell dissociation, sequencing, and mapping processes. We optimized our analysis pipeline and decided to apply three unique molecular identifiers (UMIs) as a threshold for the number of UMIs for each iTF (see Methods) (Supplemental Fig. S2B). As a result, we successfully detected 77 iTFs (7938 cells), 76 iTFs (5714 cells), and 68 iTFs (7902 cells) from day 1, 3, and 5 samples, respectively (Supplemental Table S2). The proportion of single TF overexpressing cells identified is 62.7% (day 1), 60.9% (day 3), and 58.9% (day 5), calculated among all cells. These numbers are consistently ∼60% for cells that passed quality control on each respective day.

### iTF-seq captures changes in transcriptome mediated by single TF induction

**Figure 2.**
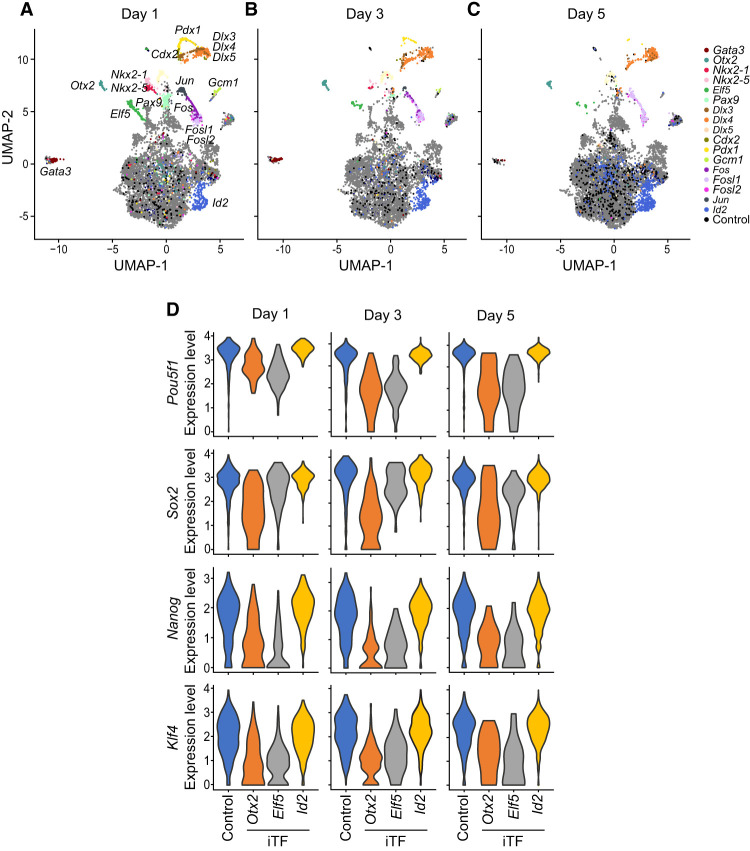
iTF-seq enables search for TFs with cell fate conversion potential. (*A*–*C*) UMAP plots of cells expressing individual iTFs and control cells after 1, 3, and 5 d of induction. Some iTF-expressing cells and control cells are marked with colors. (*D*) Relative expression levels of ESC markers (*Pou5f1*, *Sox2*, *Nanog*, and *Klf4*) in control and *Otx2*-, *Elf5*-, and *Id2*-expressing cells after 1, 3, and 5 d of induction.

With the obtained iTF-seq data, we performed UMAP clustering and visualized the positions of cells to quickly monitor the global gene expression differences among the detected cells (Fig. 2A–C). We observed one big cluster of cells along with multiple small cell clusters distinctly separated from the big cluster, indicating that the cells within the small clusters show unique gene expression profiles and are potentially differentiated owing to the induction of specific TFs with cell fate conversion potential. Indeed, as shown in Figure 2A, we found that many cells expressing iTFs with already known reprogramming potential, such as *Pdx1*, *Gata3*, and *Fosl1* (Supplemental Table S1), belong to such separated cell clusters. Notably, in contrast to many reprogramming processes characterized by low efficiencies, we observed that TFs inducing cells to locate in separated cell clusters are potent, as most such TF-overexpressing cells tend to position in separated cell clusters rather than in the large undifferentiated cell cluster. For instance, on day 1, all 93 (100%) ectopic *Dlx3*-expressing cells are localized within the separated cluster. Similarly, among 170 *Elf5*-expressing cells and 75 *Fosl2*-expressing cells, 97.8% and 80%, respectively, are found in separated clusters (Supplemental Table S3). The high efficiency of cell fate changes by inducing TFs was also observed in a few validation cases in the recent scRNA-seq-based single TF overexpression screen (>80% efficiency confirmed through testing reporter gene expression) (Ng et al. 2021). We found many additional cell clusters expressing iTFs without previously known cell fate conversion potential. This implies that iTF-seq is sensitive enough to identify previously known and unknown TFs capable of cell fate changes. As expected, single-cell transcriptome and pseudobulk RNA-seq analysis indicated that the cells belonging to the separated clusters express a relatively lower expression level of ES cell marker genes, such as *Nanog* and *Sox2*, compared with the control cells, owing to the loss of ES cell identity after iTF induction (Fig. 2D; Supplemental Table S4).

### TF induction can rapidly trigger differentiation of ES cells

None of the prior TF overexpression studies monitored time-dependent transcriptome changes (Parekh et al. 2018; Nakatake et al. 2020; Ng et al. 2021; Joung et al. 2023), and cell fate changes were monitored upon 4–7 d of TF overexpression. We presume that time course experiments would give us additional insights into the roles of TFs with cell fate conversion potential as we observed morphological changes upon 1 d of TF induction in multiple individual cases (Supplemental Fig. S2A). The UMAP visualization also found that numerous TFs could change cell fates after only 1 d of induction, suggesting that TF-mediated reprogramming can occur rapidly. Considering the typically slow reprogramming processes, such as somatic cell reprogramming, this finding shed light on the speed of TF-mediated reprogramming (Fig. 2A). It is worth noting that previous studies have suggested extremely rapid and dynamic control of TF-mediated transcriptional activity in response to a variety of stimuli, as seen in the case of the Jun–Fos AP1 complex (Devary et al. 1991; Patel et al. 1994). This further highlights the potential for speedy cell fate manipulation through TF induction.

### Determining cell fate changes by calculating the differentiation index

Although we observed that the induction of multiple TFs triggered the differentiation of ES cells, the interpretation based on the UMAP results was subjective (Fig. 2A–C). To determine changes in cell fate, we developed a quantitative measure called the “differentiation index.” We calculated the distances between the centroid of control cells and each control cell, using gene expression values as elements of the cells’ expression vectors. Next, we measured the distances between the centroid of control cells and each cell population expressing a specific iTF and normalized them based on the mean and standard deviation of the control cells. To account for the interdependence of gene expressions, we applied PCA to the expression values before measuring distances from the centroid of control cells and used coefficients of PCs for the calculation (Supplemental Fig. S3A).

**Figure 3.**
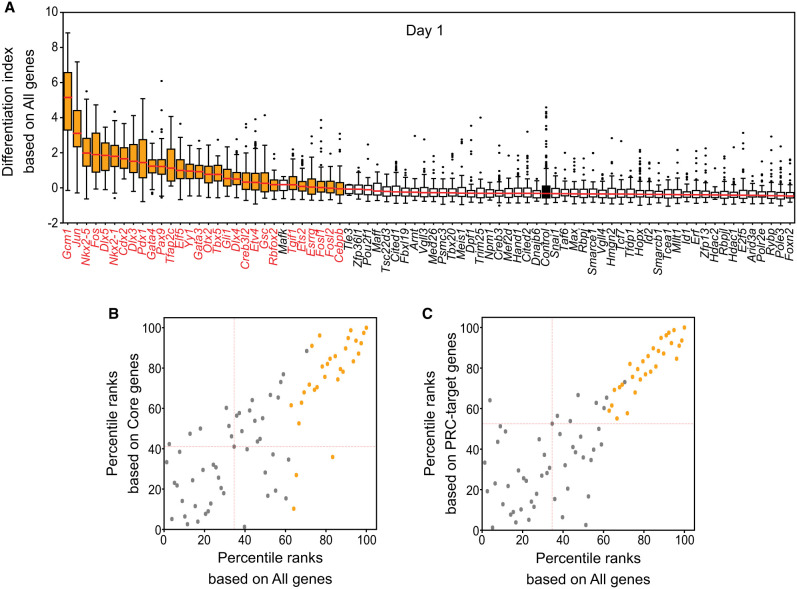
Determining cell fate changes by calculating differentiation index. (*A*) Differentiation index for cells expressing single iTF and control cells. Each box plot shows the distribution of the differentiation index for each cell population expressing one iTF (medians are marked with red lines). TFs with cell fate conversion potential are marked with orange, and the control is black. *Mafk* was not significant based on adjusted *P*-value. (*B*,*C*) Comparison between differentiation indexes based on all detected genes (all genes) and core pluripotency genes (core genes; *B*) or PRC target genes (*C*) after 1 d of induction (because the differentiation indexes for each gene group have different ranges, percentile ranks were used). TFs with cell fate conversion potential are marked with orange. Red dashed lines show the location of control cells. Some TFs (gray dots), like *Mafk*, are located near or even in the cluster of TFs with cell fate conversion potential (orange dots). These TFs were not selected based on the results of statistical testing.

To assess cell fate changes, we calculated the differentiation index using the expression data of all genes detected by iTF-seq. As expected, many cell populations with specific iTF induction showed higher differentiation index values than did the control cells (Fig. 3A), indicating significant differences in overall transcriptomes. TFs that caused such disturbances were deemed potent TFs with the ability to induce cell fate conversion. Consistent with the UMAP data (Fig. 2A), such factors include *Gcm1*, *Jun*, *Nkx2-5*, *Fos*, *Dlx5*, and *Cdx2* (Fig. 3A). We also calculated the differentiation index for the time course data and found that 29, 20, and 21 TFs corresponding to each day had cell fate conversion potential (Mann–Whitney *U* test, adjusted *P*-value < 0.01) (Supplemental Table S5). In contrast, cells with induction of many other individual TFs, such as *Cited1*, *Cited2*, *Erf*, *Hmgn2*, *Hopx*, *Id2*, *Rbpjl*, or *Snai1*, had similar or relatively lower differentiation index values compared with those of the control cells, indicating that these TFs did not significantly alter cell fates upon induction (Fig. 3A).

We further assessed the changes in expression of the extended core pluripotency-related genes, which are typically down-regulated upon differentiation of ES cells, and the polycomb repressive complex (PRC) target genes, which are up-regulated upon differentiation (Kim et al. 2010). We used these two gene sets to calculate differentiation indexes, and as shown in Figure 3, B and C, and Supplemental Figure S3B, the percentile ranks based on the index values using all genes displayed a strong positive correlation with the ranks calculated using the core-related genes (Fig. 3B) or PRC target genes (Fig. 3C). This provides independent confirmation that the potent TFs induced cell fate changes from ES cells to differentiated cells by down-regulating the ES cell core factors and activating various lineage marker genes repressed by PRC in self-renewing, undifferentiated ES cells.

### Inducing TFs for a brief duration is sufficient for cell fate changes

A common feature observed during reprogramming or transdifferentiating factors is the activation of the endogenous expression of the induced factors. To investigate whether factors with a high differentiation index activate their endogenous expression, we examined the activation of endogenous TF upon the ectopic expression. We used specific primer pairs designed to amplify the junction between the 5′ or 3′ UTR and exons for the detection of endogenous transcripts. As shown in Supplemental Figure S4A, 13 out of 18 TFs tested with a high differentiation index showed the activation of endogenous TFs. In contrast, all tested TFs with a low differentiation index were confirmed to show no activation of endogenous TFs upon induction, despite showing comparable ectopic induction levels to TFs with a high differentiation index (Supplemental Fig. S4A,B).

We investigated, aligned with examining reprogramming or cell fate–changing potential, whether differentiated cells induced by potent TFs can maintain their altered cell states without reverting to ES cell–like states or require continuous Dox treatment. To address this, we conducted a time course transcriptome profiling of Dox removal samples for potent TFs, including *Dlx5*, *Gata3*, *Gcm1*, *Otx2*, and *Pdx1*. In addition to maintaining continuous Dox treatment for 7 d as a control, we generated samples by discontinuing Dox after 1 day, 3 days, and 5 days of culture. As shown in Supplemental Figure S4C, visualizing 3000 genes showing higher expression variance, the overall gene expression patterns after Dox removal largely differ from those of control ES cells and align more closely with Dox-maintained cell lines for 7 d. In most instances, our findings indicate that a 3-d period of TF induction via Dox treatment is sufficient to commit cells to differentiated states, with no subsequent regaining of ES cell line expression patterns. We observed that even 1 d of induction followed by extended culture without Dox resulted in losing an ES cell–specific transcriptome profile. To further assess the maintenance of differentiated status in potent TF-induced cells, we monitored the expression of pluripotency markers (*Pou5f1*, *Nanog*, and *Sox2*) in cells overexpressing *Yy1*, *Creb3l2*, *Cdx2*, *Ets2*, *Gsc*, or *Cebpb*, followed by treatment with Dox for 1 or 5 d. As shown in Supplemental Figure S4D, the expression of pluripotency marker genes decreased over time compared with their levels in control ES cells, albeit with variable patterns. In summary, there was no substantial evidence of cells returning to ES cell–like states over time.

### ES cells experiencing fate changes show slower proliferation and increased cell death

**Figure 4.**
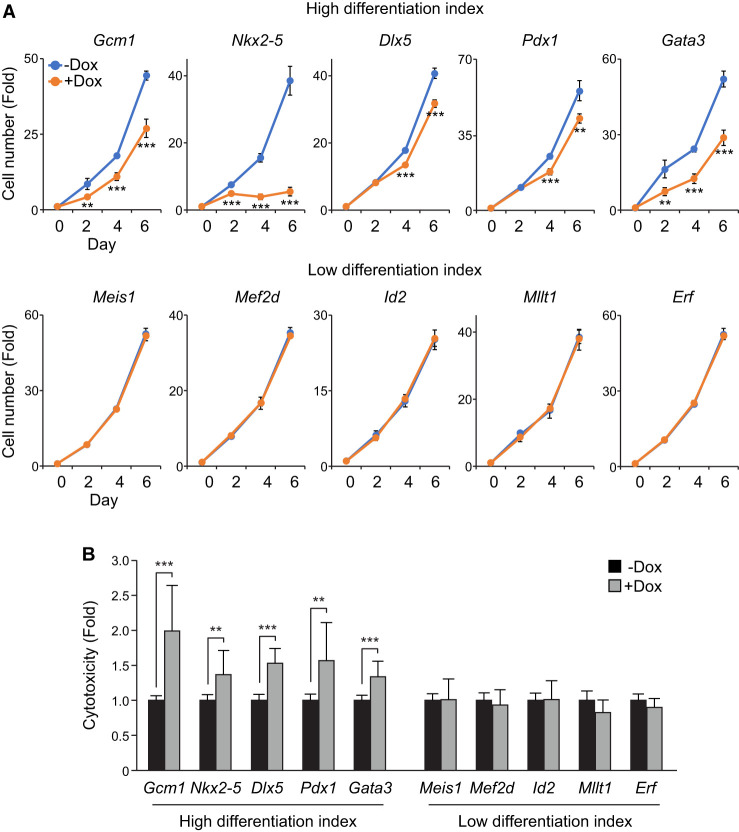
ES cells experiencing fate changes show slower proliferation and increased cell death. (*A*) Line plots depicting the time course measurement of cell number upon overexpression of iTFs via Dox induction (0.5 µg/mL; +Dox). The untreated cells (−Dox) were used as a control. The *top* row represents iTFs with high differentiation indexes, and the *bottom* row represents iTFs with low differentiation indexes. Cell numbers were counted up to 6 d after Dox induction, and the fold changes in cell numbers from day 0 were calculated and plotted (n = 2). Data were represented as mean ± SD, and a Student's *t*-test was performed to determine significance between −Dox and +Dox: (**) *P* < 0.01, (***) *P* < 0.001. (*B*) A bar plot shows the fold change in cell death in 10 iTF lines upon overexpression of the iTF by treating Dox (0.5 µg/mL) for 2 d. The cytotoxicity was calculated by measuring lactate dehydrogenase (LDH) activities in the culture media. The percentage of cytotoxicity was calculated for each value by dividing it against the value from the wells treated with lysis buffer, which provided the maximum LDH activity. The fold change was calculated against the control samples. Finally, the average percentage of cytotoxicity was calculated across replicates (n = 3–6), and a Student's *t*-test was performed to determine significance: (**) *P* < 0.01, (***) *P* < 0.001.

We observed that the proportion of some iTF-induced cell populations forming separate UMAP clusters, including *Pdx1*, *Cdx2*, *Gata3*, and *Gcm1*, gradually decreased with longer induction durations (Fig. 2A–C; Supplemental Table S2). Because ES cells have a higher proliferation rate than differentiated cells (Zaveri and Dhawan 2018) and we maintained leukemia inhibitory factor (LIF) in the culture even after iTF induction, the dilution of iTF-induced cells by rapidly proliferating ES cells over time is likely responsible for this observation. Therefore, we hypothesized that some TFs, barely detected in day 3 or 5 samples, such as *Pdx1*, *Jun*, *Nkx2-5*, and *Gata3*, might still have significant reprogramming potential (Supplemental Fig. S5A; Supplemental Table S2). Additionally, as pluripotent stem cells undergo increased cell death upon differentiation (Duval et al. 2000; Bashamboo et al. 2006), we thought that cell death might also contribute to the decreased proportion of some TF-induced cells. To address these possibilities, we monitored the proliferation rate and cell death of multiple individual iTF lines associated with a high differentiation index (i.e., *Gcm1*, *Nkx2-5*, *Dlx5*, *Pdx1*, and *Gata3*) and a low differentiation index (i.e., *Meis1*, *Mef2d*, *Id2*, *Mllt1*, and *Erf*) in Figure 3A. Our results validated that cells with a high differentiation index, indicating rapid differentiation, show decreased proliferation with increased cell death, whereas the cells with a low differentiation index show no significant changes in cell proliferation and cell death (Fig. 4A,B; Supplemental Fig. S5B,C). Hence, our conclusion is that the rates of proliferation and cell death are intricately connected to TF-mediated differentiation of ES cells. The findings further indicate that when assessing differentiated or differentiating ES cells, proliferation and cell death rates should be regarded as pivotal factors in high-throughput differentiation screens. Moreover, these results underscore the advantages of using controlled TF induction, an approach not attainable with constitutive promoter-based methods.

### iTF-mediated rapid global expression changes and lineage specifications

Using the Dox-inducible system in our approach allowed us to reveal that a mere 1-d induction of potent TFs is sufficient to alter the global gene expression profile (Fig. 2). To delve deeper into the speed of cell fate changes, we conducted global gene expression profiling at additional time points, 3 h, 6 h, 12 h, day 1, day 3, and day 5, following the induction of *Otx2*, *Gcm1*, or *Dlx5*. As shown in Supplemental Figure S6A, although the timing varies depending on the iTFs, a 12-h induction is sufficient to transform the transcriptome from that of uninduced ES cells, suggesting that iTF-mediated cell fate changes are exceptionally rapid and dynamic processes.

**Figure 5.**
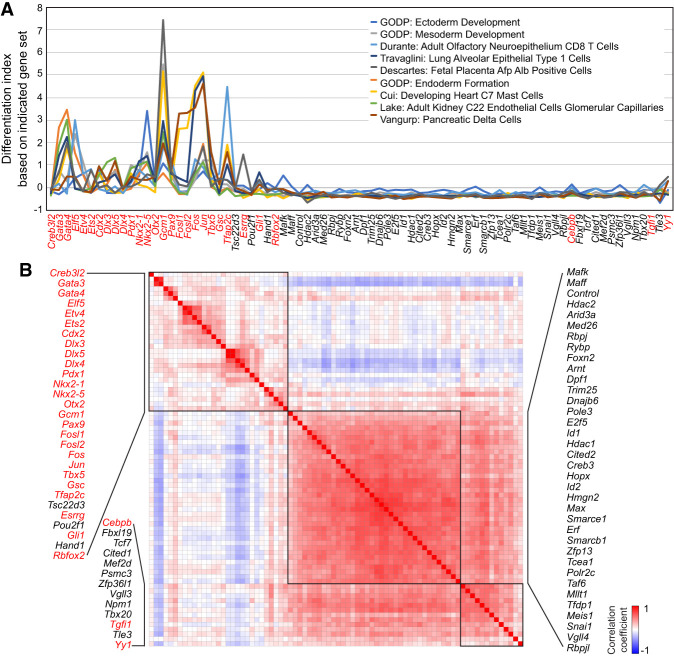
iTF-mediated lineage specification. (*A*) Differentiation index calculated with gene expression responses to the induction of 80 individual TFs (day 1) and indicated gene sets obtained from MSigDB signature gene set collection. Red text indicates TFs harboring cell fate conversion potential defined by differentiation indexing (Fig. 3A). (*B*) Similarity matrix of iTFs calculated from the differentiation indexes, which were based on all pairs of iTFs and 643 gene sets from MSigDB. Some groups of TFs show high similarity and form clusters of red cells and black boxes, meaning they have similar differentiation indexes across various cell type– or lineage-specific gene sets. Red text indicates TFs with cell fate conversion potential defined by differentiation index.

To further investigate the specific lineages induced by each TF induction, we calculated differentiation indexes using 643 additional gene sets from the Molecular Signature Database (MSigDB), including three germ layer–related gene sets (total, eight: two from C2 curated and six from C5 ontology gene set collection) and C8 cell-type signature gene set collection (Subramanian et al. 2005; Liberzon et al. 2011). Overall, TFs with high differentiation index values based on all genes detected from our samples (Fig. 3A) also show relatively high values from the test of MSigDB gene sets (Supplemental Table S6), and some cases indicated incipient trajectories of early differentiation. For instance, *Gata4*- and *Gata3*-induced cells showed the highest values from endoderm gene sets, whereas *Elf5*-induced cells showed high values from mesoderm gene sets. Moreover, we found that *Gcm1* overexpression induces trophoblast or placenta-related cell types, and *Jun*, *Fos*, or *Gcm1* induction generates heart-related cell types. Notably, some TFs, such as *Jun*, *Fos*, and *Gcm1*, activated gene sets associated with multiple lineages or specialized cell types (Fig. 5A; Supplemental Table S6). We also computed a similarity matrix with the median values of differentiation indexes for all available pairs of gene set groups and iTFs (Fig. 5B) and found that among TFs with cell fate conversion potential, those belonging to the same family often showed similar lineage specification potential, indicating either redundancy or positive feedback loops among these family TFs (e.g., GATA3 and GATA4; DLX3, DLX4, and DLX5; NKX2-1 and NKX2-5; and AP-1 complex proteins, including JUN, FOS, FOSL1, and FOSL2). To validate the induced lineage markers associated with cells overexpressing TF within the same family, we individually overexpressed each TF using iTF lines. Subsequently, we monitored the expression of associated lineage marker genes through reverse transcription quantitative polymerase chain reaction (RT-qPCR). Marker genes for distinct cell types/lineages were selected from MSigDB, PanglaoDB (Franzén et al. 2019), and the Azimuth data set (Hao et al. 2021). As illustrated in Supplemental Figure S6B, the induction of *Dlx3*, *Dlx4*, or *Dlx5* led to increased expression levels of marker genes associated with fetal lung ciliated epithelial cells, as indicated by a high differentiation index calculated using the Descartes fetal lung ciliated epithelial cell gene set (Supplemental Table S6). We also confirmed the common activation of lineage marker genes for fetal spleen vascular endothelial in *Nkx2-1* and *Nkx2-5* overexpressed cells (Supplemental Table S6; Supplemental Fig. S6C). In addition to using the differentiation index, we used SingleCellNet (Tan and Cahan 2019) in conjunction with training data (Han et al. 2018) to monitor iTF-mediated lineage specification. However, we encountered challenges in predicting potential lineages, indicating that SingleCellNet might not be optimal for analyzing our data, possibly owing to the early differentiation status of the iTF cells.

### GCM1 and OTX2 function as activators during TF-mediated cell fate changes

Among genes that encode TFs that rapidly trigger cell fate changes, *Gcm1* and *Otx2* are of particular interest, as their cell fate conversion potential has not been reported in mouse ES cells. *Gcm1* had the highest differentiation index among all potent TF genes (Fig. 3A) and is expressed explicitly in a subset of mouse placental trophoblast cells, in which it plays a critical role in placental cell fusion (Stecca et al. 2002). Our analysis of pseudobulk RNA-seq data and independent RT-qPCR confirmed that *Gcm1*-induced cells express many trophoblast marker genes, such as *Gata3*, *Arid3a*, *Hand1*, *Tfap2c*, and *Cdx2* (Supplemental Fig. S7A; Supplemental Table S4), suggesting that *Gcm1 trans*-differentiates ES cells to trophoblast-like cells. *Otx2*, on the other hand, encodes a TF previously implicated in brain, cerebellum, and eye development (Ruiz-Reig et al. 2019), and a high level of *Otx2* induces rod cell fate in retinal progenitors (Yamamoto et al. 2020). Recent screens also revealed that overexpression of human *OTX2* triggers differentiation of human pluripotent stem cells (Ng et al. 2021; Joung et al. 2023). We observed that *Otx2* induction activates the expression of multiple neuronal lineage markers in both pseudobulk RNA-seq data and RT-qPCR analysis, consistent with its in vivo function (Supplemental Fig. S7B; Supplemental Table S4).

**Figure 6.**
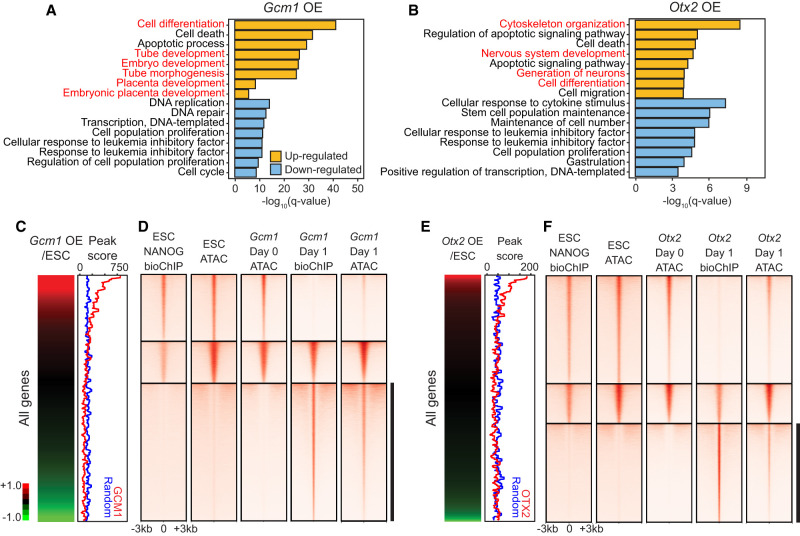
GCM1 and OTX2 function as activators and pioneer factors. (*A*,*B*) Selected GO biological process terms significantly enriched among differentially expressed genes up-regulated or down-regulated in *Gcm1*-overexpressing cells (*A*) or *Otx2*-overexpressing cells (*B*). (*C*,*E*, *left*) Expression profiles of *Gcm1*-overexpressing cells (*C*) or *Otx2*-overexpressing cells (*E*) with genes ordered by their expression levels in *Gcm1/Otx2*-overexpressing cells relative to the control ES cells. (*Right*) Corresponding peak scores to the expression profiles (red) and randomized peak scores (blue) are shown by applying moving window average (window size, 100; bin size, one). (*D*,*F*) bioChIP-seq of indicated TFs and ATAC-seq signals detected in control ES cells, −Dox cells (day 0), and *Gcm1*-overexpressing cells (*D*) or *Otx2*-overexpressing cells (*F*; day 1). Black bars indicate the regions closed in ES cells or day 0 cells but occupied by GCM1 (*D*) or OTX2 (*F*) and become open.

To further investigate the cell fate–changing capacity of *Gcm1* and *Otx2*, we analyzed the pseudobulk RNA-seq data and identified differentially expressed genes upon the induction of each TF. As shown in Figure 6, A and B, Gene Ontology (GO) analysis revealed that the down-regulated genes upon the induction of *Gcm1* (491 genes) or *Otx2* (176 genes) are associated with ES cell maintenance and proliferation-related terms, indicating that both *Gcm1* and *Otx2* triggered the differentiation of ES cells upon induction. The up-regulated genes by *Gcm1* (1806 genes) were enriched in differentiation-related terms, including cell differentiation, tube development, and placenta development in accordance with the up-regulation of trophoblast marker genes. The 257 genes up-regulated in *Otx2*-induced cells were related to nervous system development, which is in accordance with previous reports (Ruiz-Reig et al. 2019). Overall, induction of *Gcm1* and *Otx2* resulted in cell fate conversion by down-regulation of ES cell marker genes and promoting the activation of lineage-specific genes.

As the SBFB vector used in iTF-seq enables biotinylated iTF-mediated mapping of PDI and PPI (Kim et al. 2009), we aimed to gain insight into the molecular mechanisms underlying the cell fate changes induced by *Gcm1* and *Otx2*. Therefore, we performed biotin-mediated chromatin immunoprecipitation followed by high-throughput sequencing (bioChIP-seq) to identify direct targets of these TFs (Supplemental Fig. S7C). As both TFs rapidly induced cell fate changes, we performed bioChIP-seq after 1 d of induction. bioChIP-seq analysis revealed 130,265 and 88,585 target peaks of GCM1 and OTX2, respectively, and motif analysis using HOMER (Heinz et al. 2010) showed that the top-ranking motifs of GCM1 and OTX2 were similar to their previously known motifs (Supplemental Fig. S7D). Both TFs showed a strong preference for binding to distal enhancers, which are known to be strongly associated with cell fate determination (Supplemental Fig. S7E; Ong and Corces 2012). Furthermore, a combined analysis of bioChIP-seq and pseudobulk RNA-seq data showed that both TFs had a strong occupancy on the genes activated by the induction of each TF (Fig. 6C,E). These results suggest that GCM1 and OTX2 function as transcriptional activators during cell fate conversion.

### GCM1 and OTX2 function as pioneer factors

Because the genes directly targeted by GCM1 and OTX2 during TF-mediated cell fate conversion were not generally active in self-renewing ES cells (Fig. 6C,E), we hypothesized that GCM1 and OTX2 might have pioneer factor activity (Iwafuchi-Doi and Zaret 2016; Morris 2016), which would allow them to bind to and open up previously inaccessible chromatin regions. To test this, we analyzed the chromatin accessibility of ES cells before and after induction of each TF using assay for transposase-accessible chromatin with high-throughput sequencing (ATAC-seq). As shown in Figure 6, D and F, we found that the chromatin accessibility of control NANOG binding sites was similar to those in ES cells and the cells without TF induction. However, subsets of GCM1 and OTX2 target loci displayed extremely low ATAC-seq signals in control ES cells or the cells without induction, but their ATAC-seq signals increased significantly upon induction of GCM1 and OTX2, suggesting that these TFs likely act as pioneer factors and increase the chromatin accessibility of silenced lineage markers in ES cells, thereby facilitating cell fate conversion.

## Discussion

In summary, this study highlights the potential of iTF-seq to identify previously unknown potent TFs with cell fate conversion potential. In addition, the iTF-seq approach provides a powerful tool for investigating the molecular mechanisms of cell fate conversion induced by TFs as we found that GCM1 and OTX2 act as pioneer factors to induce cell fate conversion by directly activating lineage-specific genes residing in regions of closed chromatin. Future studies using iTF-seq could focus on identifying additional TFs involved in cell fate conversion and investigating their specific mechanisms of action, such as potential roles as repressors or dual-function TFs. Additionally, mapping the interaction partner proteins of the TFs with the iTF-seq pipeline can provide further insights into their unique features and modes of action. Overall, iTF-seq has the potential to significantly advance our understanding of the complex molecular events underlying cell fate conversion.

Many previous studies have used multiple TFs for cell fate reprogramming. However, emerging research revealed that even a single TF can induce cell fate conversion toward a specific lineage or cell type, including recent large-scale screens (Parekh et al. 2018; Joung et al. 2023). By using appropriate culture conditions, like the somatic cell reprogramming case, a single TF-mediated cell fate change can be further polished for generating functional cell types. These findings highlight the merits of a single-factor system, particularly when integrated with downstream approaches aimed at understanding the action mechanisms of potent individual TFs, as elucidated in this study. Testing multiple TFs based on the results obtained from a single TF screening could enhance the efficiency of cell fate conversion.

Our observations on the decreased proportion of cells expressing TFs capable of triggering cell fate changes during prolonged induction are consistent with previous reports on the differences in cell cycle phase duration and cell death between pluripotent stem cells and differentiated cells. It is important to note that 20%–30% of mouse ES cells undergo cell death upon exit from self-renewal (Duval et al. 2000; Bashamboo et al. 2006), which may also contribute to cell fate conversion upon TF induction. In addition, we observed that the decreased expression levels of pluripotency markers persisted in cells overexpressing the iTFs, such as *Yy1*, *Creb3l2*, *Cdx2*, *Ets2*, *Gsc*, and *Cebpb*, which showed cell fate–changing effects only evident in day 1 results. This observation suggests that the loss of these cells on days 3 and 5 did not result from a reversion to ES cell–like states over time but may stem from other reasons. We confirmed that multiple iTF-triggered differentiated cells show increased cell death. Therefore, we conclude that both the slower cell cycle and enhanced cell death upon differentiation are responsible for the decreased proportion of cells observed during more prolonged induction of potent TFs. Our discoveries underscore the significance of considering cell cycle and cell death in perturbation screens related to the differentiation of ES cells. This aspect has not been addressed in previous screens that lacked monitoring dynamic changes in cellular status through an inducible system. This understanding will be essential in designing more effective screening strategies for identifying potent TFs capable of inducing cell fate conversion.

The SB approach induces a spectrum of ectopic TF expression levels and a heterogeneous cell population, which may affect cell fate–changing efficiency. We observed a range of TF expressions among iTF overexpression cells (Supplemental Fig. S8A). The variability in ectopic TF levels may be attributed to the number of inserted iTFs and potential positional effects. In our observations, cells showing higher ectopic TF expressions (top 33%) tended to display relatively lower expression of ES cell markers (*Pou5f1*, *Sox2*, and *Nanog*), whereas cells with lower TF expression (bottom 33%) showed somewhat higher levels of ES cell marker genes compared with cells expressing high levels of iTF. It is noteworthy that in both cases, the levels of ES cell markers were lower than those in undifferentiated control ES cells, indicating the ES cell differentiation. Although we acknowledge that this variability in ectopic TF levels introduces noise into the system, we recognize its potential to offer valuable insights into TF dosage effects. We unexpectedly observed that the induction levels of *Gata3* influence lineage specification decisions toward trophoblast or primitive endoderm lineages. Cells with high *Gata3* expression displayed trophoblast-like gene expression programs, whereas those with low *Gata3* expression expressed primitive endoderm-like gene expression patterns (Supplemental Fig. S8B). Consequently, although our system may generate complex expression patterns contingent on the levels of ectopic TFs, we posit that these resultant patterns may offer an additional layer of information for a deeper understanding of TF dosage effects in cell fate conversion, a facet not easily gleaned from other methodologies.

Although some tissue/cell type–specific TFs activated the expected tissue/cell type–specific genes upon induction, we also observed that several TFs activated various lineages or a broad spectrum of gene sets. Notably, we found that the induction of multiple family TFs resulted in similar transcriptome changes (Fig. 5B; Supplemental Fig. S6B,C). Especially, ectopic induction of all tested *Jun/Fos* family TFs increased the expression levels of marker genes associated with multiple lineages, although each factor showed distinct kinetics. Although FOS and JUN showed rapid activation of lineage marker genes, with expression evident in day 1 samples, *Fosl1*- and *Fosl2*-overexpressing cells displayed a noticeable activation of lineage marker genes after 3 d of induction. Therefore, although their potency and target activation timing may vary, the immediate early genes, *Jun* and *Fos*, along with their family proteins, *Fosl1* and *Fosl2*, activated more than two lineages, suggesting that these TFs can activate multiple lineages (Supplemental Fig. S6D). Although not tested, our current experimental setting suggests that multiple family TFs generating similar transcriptome changes might have similar chromosomal target genes. As each TF within the same family often shows distinct cell type–specific expression patterns, our approach may help to elucidate the context-dependent roles of family TFs by testing additional cell types. Investigating whether these TFs occupy similar genomic targets would be of great interest in understanding the potential and general behavior of individual family TFs.

Our study showed that both GCM1 and OTX2 possess pioneer factor activity by binding closed chromatin in ES cells, leading to the opening of the target loci. It remains to be tested whether other potent TFs we identified also have pioneer activity and can contribute to generating open chromatin. Understanding the requirement of pioneer factor activity for TF-mediated cell fate conversion will be essential to uncover the underlying mechanisms of this process. Although GCM1 and OTX2 induced distinct gene expression profiles upon induction, it is possible that they share common interaction partner proteins, such as the factors involved in enhancer regulation, enhancer-related histone signatures, and chromatin remodeling, in addition to their unique interaction partner proteins. Therefore, identifying the interaction partner proteins of potent TFs will be crucial to understand both general and unique mechanisms of TF-mediated cell fate conversion. Our iTF-seq approach, which is also compatible with the biotinylation-mediated immunoprecipitation followed by mass spectrometry (bioIP/MS) approach (Kim et al. 2009), will provide a powerful platform to identify TFs capable of cell fate changes and to gain insights into the mechanisms of TF-mediated cell fate conversion. This approach holds great potential for generating desired cell types and for advancing our understanding of cell fate conversion processes.

Although we successfully identified potent cell fate–changing TFs that showed rapid effects based on day 1 results, the current method also presents limitations concerning the availability of informative cells (differentiated) at later time points, necessitating significantly more sequencing depth. Therefore, incorporating a depletion step to exclude undifferentiated cells, such as by using SSEA-1 expression, could enhance the efficiency of identifying potent cell fate–changing TFs using iTF-seq. Additionally, the SB transposon system integrates varying numbers of iTFs into each cell, resulting in a spectrum of TF expression levels among iTF lines. Although this variability in ectopic TF levels introduces noise into the system, it simultaneously provides valuable insights into TF dosage effects.

## Methods

### Cell culture

All the ES cell lines, including BirA-expressing mouse J1 ES cells (BirA-ES cells) and stable inducible lines, were cultured on 0.1% gelatin-coated plates in Dulbecco's Modified Eagle Medium (DMEM) (high glucose, Gibco 11965092) supplemented with 18% fetal bovine serum (Gemini Bio-Products 100106), 100 µM MEM nonessential amino acids (100X stock, Gibco 11140050), EmbryoMax nucleosides (MilliporeSigma ES-008-D), 2 mM L-glutamine, 50 U/L penicillin–streptomycin (100X stock, Gibco 10378016), 100 µM β-mercaptoethanol, and 1000 U/mL mouse LIF (Gemini Bio-Products 400–495) in a 37°C incubator with 5% CO_2_. Cells were passaged every 2 d by dissociating into single cells with 0.25% trypsin-EDTA.

### Construction of pSBFB plasmid and generation of stable inducible overexpression cell lines

The luciferase ORF of pSBtet-GP (Addgene 60495) was replaced by a FLAG sequence, biotinylation site, and multiple cloning sites with NotI and NheI sequences (pSBFB). For cloning, total RNA was extracted from mouse J1 ES cells using the RNeasy plus mini kit (Qiagen 74136). Then, the RNA was reverse-transcribed with the ProtoScript II first strand cDNA synthesis kit (New England Biolabs [NEB] E6560). Each gene of interest was amplified from the cDNA and cut with NotI and/or NheI depending on the presence of respective cut sites within the ORF. Cloning was performed using T4 DNA ligase (NEB M0202) using pSBFB digested with NotI and/or NheI enzymes. The cloned vectors, verified with Sanger sequencing, were transfected (lipofectamine 3000, Thermo Fisher Scientific L3000008) into BirA-ES cells (Kim et al. 2008) along with pCMV(CAT)T7-SB100 (Addgene 34879), conferring transient expression of the SB transposase for inserting the target sequence into the genome of BirA-ES cells. To obtain stable cell lines, the transduced cells were selected using puromycin (1 µg/mL, Gibco A1113803) and G418 (250 µg/mL, Gibco 10131027) at 24 h after transfection. Protein expression of the transfected gene of each cell line was confirmed with western blotting after treatment with Dox (Fisher BioReagents BP26535) for 24 h at a concentration of 0.5 μg/mL.

### Western blotting

Dox-treated or untreated cells were lysed using Laemmli sample buffer (Bio-Rad 1610747) supplemented with 5% β-mercaptoethanol (MilliporeSigma M3148) and heated for 10 min at 95°C. The cell lysate was run on sodium dodecyl sulfate (SDS) polyacrylamide gel and transferred onto polyvinylidene fluoride (PVDF) membranes (MilliporeSigma IPVH00010). Then, membranes were blocked with either 5% nonfat milk or BSA in TBS-T (Tris-buffered saline with 0.1% Tween-20) for 1 h. Subsequently, the membranes were incubated with streptavidin–horseradish peroxidase conjugates (streptavidin-HRP, Cytiva RPN1231, 1:2000) or ACTB antibody (Abgent AM1829B, 1:20,000). For ACTB detection, the membrane was incubated with a secondary antibody (Cell Signaling Technology 7076, 1:10,000) for 1 h at room temperature. The membrane was then exposed to ECL substrate (Cytiva RPN2232), and visualized on a ChemiDoc XRS+ imaging system (Bio-Rad).

### RT-qPCR

Total RNA was extracted using RNeasy plus mini kit (250, Qiagen 74136). For each sample, 500 ng RNA was reverse-transcribed with qScript cDNA supermix (Quantabio 95048-100). cDNA was diluted into 200 μL, and qPCR was performed using PerfeCTa SYBR Green FastMix (QuantaBio 95072-012). The primers used for qPCR are listed in Supplemental Table S7. Results were calculated from three independent samples (n = 3), and statistical significance was determined using two-tailed Student's *t*-test analysis.

### Immunofluorescence

Immunofluorescence was performed on *Gcm1* and *Otx2* SBFB cells after 24 h of Dox induction with uninduced control cells. Cells were washed twice with PBS and fixed with freshly cracked 4% paraformaldehyde in PBS for 20 min at room temperature. After washing three times with wash buffer (0.1% BSA in PBS), the cells were blocked in blocking buffer (PBS containing 0.1% BSA, 10% horse serum, and 0.3% Triton X-100) for 45 min. The cells were then incubated with streptavidin, Alexa Fluor 555 conjugate (Thermo Fisher Scientific S21381; diluted at 1:500 in PBS with 0.1% BSA and 10% horse serum) overnight at 4°C, followed by three washes with wash buffer. After incubation with DAPI for 5–10 min, the cells were rewashed and visualized under a fluorescence microscope.

### Pooled induction of target genes and scRNA-seq

In total, 80 iTF lines that were confirmed to express the protein of interest were pooled and cultured in ES cell media. Pooled cell lines were then treated with Dox (0.5 μg/mL) for 1, 3, and 5 d. At each time point, Dox-treated and control ES cells were dissociated into single cells with 0.25% trypsin-EDTA. The dissociated cells were washed twice with 0.04% BSA in PBS and resuspended to a concentration of 1000 cells per microliter. Then the cells were subjected to scRNA-seq. Single-cell libraries were generated using the chromium next GEM single-cell 3′ library kit v3.1 (10x Genomics), and the libraries were sequenced on a NovaSeq 6000 (Illumina).

### Sequencing read alignment and cell quality control

Sequencing reads were aligned with Cell Ranger v6.0.1 (Zheng et al. 2017). The mouse 2020-A reference built by 10x Genomics, which is based on Ensembl release 98 (Yates et al. 2020) GRCm38 genome assembly and GENCODE M23 annotation (Frankish et al. 2019), was used as a reference sequence. Cell quality control was performed with Seurat v4.0.3 (Hao et al. 2021). Based on plots illustrating the distribution of quality-control metrics, the criteria for filtering of cells were determined: A cell should have more than 1000 unique detected genes, with <10% of reads mapped to the mitochondrial genome, and more than 10,000 detected molecules. After filtering, 65%–77% of cells were selected (R Core Team 2021).

### Identification of cells overexpressing TFs

We defined a forward tag as 21 bp from a TF's start codon and defined its upstream tag as 7 bp from the vector. For the reverse tag, we concatenated a TF's last 21 bp (including the stop codon) with the downstream 7 bp from the vector and took its reverse complement. These tags were unique for each TF and allowed us to distinguish between the wild type and induced version of each gene. The only exception was the Dlx4 forward tag, which was 30 bp long because the 28-bp tag completely aligned to the reference genome.

BAM files were converted to FASTA files using SAMtools v1.7 (Li et al. 2009). Reads without either cell barcodes or UMIs were removed. Then, BLAST was performed to detect reads aligned with the tags. Collected reads with the tags were organized to the transcript level. The alignment results based on our tags were also compared with the alignment by Cell Ranger. When more than half of a transcript's reads aligned to two different genes by the two methods, that transcript was excluded. We did not exclude the cases in which a gene was annotated as “NA” by Cell Ranger but aligned with our tags and used the alignment results for further analysis.

### Adjustment of minimum UMI thresholds and cell selection

Because the iTF lines were generated individually, the number of unique iTFs expressed in a cell is theoretically one. However, errors from sequencing and library preparation steps such as polymerase chain reaction (PCR) can introduce errors that may lead to incorrect iTF prediction (Stoler and Nekrutenko 2021). To reduce false-positive events in detecting iTFs, we exploited UMIs. We applied different thresholds for the minimum number of UMI (one to five) and counted cells with single iTF detection. As the number of cells with a single iTF peaked in the minimum UMI 3 threshold, this threshold was adopted to designate iTF cells. The control cells were identified by excluding cells expressing any iTFs with a minimum UMI threshold of one.

### UMAP visualization of iTF-seq data

Uniform manifold approximation and projection (UMAP) plots were drawn with cells overexpressing a single iTF and control cells using Seurat v4.0.3 (Hao et al. 2021). Expression values were normalized and variance-stabilized by SCTransform (Hafemeister and Satija 2019). The location of whole cells was visualized, and TF-overexpressing cells for each TF and control cells were marked.

### Pseudobulk RNA-seq processing

Pseudobulk RNA-seq expression values were calculated by taking the mean of gene expression values for every gene from iTF-overexpressing cells and control cells, respectively. Log-normalized versions of corrected counts by SCTransform (Hafemeister and Satija 2019) were used (Supplemental Table S4).

### Calculation of differentiation index

First, because gene expression values are not independent of each other, we performed principal component analysis (PCA) after normalizing, variance-stabilizing, and estimating missing values with SCTransform. Fifty principal components (PCs) were used because the variance explained by each PC becomes very small around the 50th PC. The centroid of control cells based on the PCs was calculated; distances between the centroid and each control cell were measured; and these distances were converted into *z*-values. Then, distances between TF-overexpressing cells and the centroid of control cells were measured and normalized using the mean and standard deviation from control cells. For these calculations, gene expression measurements for the 80 TFs were excluded.

In addition to all genes, for some analyses, differentiation indexes were calculated with other specific gene sets: core module genes, PRC module genes (Kim et al. 2010), and 643 gene sets from the MSigDB (Subramanian et al. 2005; Liberzon et al. 2011). The 80 TFs of interest were excluded from all gene sets, and ribosomal genes (Nakao et al. 2004) were additionally excluded from the two module gene sets. For gene sets from MSigDB, we chose three germ layer–related gene sets (total, eight: GO:0007398, GO:0007492, GO:0001706, GO:0035987, GO:0007498, GO:0048332, GO:0048333, and GO:0031016) from C2 curated and C5 ontology gene set collection and all the gene sets (total, 704) from the C8 cell type signature gene set collection. Mouse orthologs of human genes were listed using Ensembl BioMart (Kinsella et al. 2011), and gene sets whose size was fewer than 15 genes were filtered out. The median values of differentiation indexes were used for the hierarchical clustering of all available pairs of gene sets and iTFs (Supplemental Table S6).

### Analysis of differentially expressed genes

Global gene expression patterns of TF-overexpressing cells at each time point to their respective control cells were compared by using Seurat v4.0.3. We computed differential expression with DESeq2 (Love et al. 2014) based on raw expression values, excluding cases when the number of TF-overexpressing cells was fewer than three. Differentially expressed genes were filtered with these criteria: average |log_2_(Fold Change)| ≥ 0.5, and Bonferroni-corrected *P*-value < 0.05. iTFs themselves were not counted as up-regulated genes. The GO term enrichment tests were performed with the gprofiler2 R package (Kolberg et al. 2020).

### RNA sequencing and data analysis

Total RNA extraction was performed using RNeasy plus mini kit (250) (Qiagen 74136), and mRNA isolation was accomplished using magnetic mRNA isolation kit (oligo(dT) beads; NEB E7490). The sequencing libraries were prepared using NEBNext ultra DNA library prep kit (NEB E7645S) and were sequenced on an Illumina NovaSeq 6000 using 150-bp paired-end reads. Adapter/quality trimming was performed with Trim Galore! v0.6.10 (Babraham Bioinformatics; https://github.com/FelixKrueger/TrimGalore). Quantification of transcript expressions was performed with Salmon v1.10.2 (Patro et al. 2017), and aggregation to the gene level was performed with the R package tximport v1.22.0 (Soneson et al. 2015).

### Cell proliferation assay

iTF lines were seeded at a density of 6 × 10^4^ cells/mL in the indicated ES cell media in 0.1% gelatin-coated 24-well plates. Dox was treated 24 h after seeding at a concentration of 0.5 µg/mL. Cells were counted at four time points: on the day of Dox induction and on days 2, 4, and 6 of Dox induction, using the Invitrogen countess automated cell counter (Invitrogen). Briefly, cells were trypsinized with 0.25% trypsin-EDTA, and then, 10 µL of the cell suspension was mixed with 10 µL of 0.4% trypan blue and counted by the cell counter.

### Cell death assay

iTF lines were seeded at a density of 5 × 10^5^ cells/mL in the indicated ES cell media in 0.1% gelatin-coated 96-well plates. Dox was treated 24 h after seeding at a concentration of 0.5 µg/mL. After 2 d of Dox induction, lactate dehydrogenase (LDH) activity was quantified using the CyQUANT LDH cytotoxicity assay kit (Thermo Fisher Scientific C20301) according to the manufacturer's instruction. The 10× lysis buffer and nuclease-free water were added directly to the media and incubated at 37°C. After 45 min, 50 µL of the media was transferred to a new 96-well plate containing an equal volume of reaction mix. The plates were incubated for 30 min at room temperature in the dark. Stop buffer was added to each well, and the final absorbance was measured at 490 nm with the Tecan M1000 plate reader. The results were normalized to wells that had been treated with lysis buffer, providing the maximum LDH activity.

### bioChIP-seq and ATAC-seq

bioChIP-seq was performed on *Gcm1* and *Otx2* iTF lines with or without Dox induction, as well as ES cells expressing biotinylated NANOG. For each bioChIP reaction, 5 × 10^6^ cells were fixed using 1% formaldehyde for 7 min at room temperature, and then the fixation was stopped by the addition of glycine to a final concentration of 0.125 M. The bioChIP was performed as described previously (Kim et al. 2009). The genomic DNA was sonicated into fragments of ∼200 bp using a Bioruptor (Diagenode). The cross-linked DNA fragments were precleared using Protein A agarose (Sigma-Aldrich 11134515001) and immunoprecipitated using Dynabeads MyOne streptavidin T1 (Invitrogen 65602). The sequencing libraries were prepared using the NEBNext ultra DNA library prep kit (NEB E7645S) and were sequenced on an Illumina NovaSeq 6000 using 50-bp paired-end reads. An assay for transposase-accessible chromatin with high-throughput sequencing (ATAC-seq) was performed on iTF lines for *Gcm1* and *Otx2* under uninduced or 1-d-induced conditions. ATAC-seq libraries were generated using Diagenode's ATAC-seq kit (C01080001) followed by sequencing on Illumina NovaSeq 6000 using 50-bp paired-end reads.

### bioChIP-seq and ATAC-seq data analysis

Published ES cell ATAC-seq data were downloaded from the NCBI Gene Expression Omnibus (GEO; https://www.ncbi.nlm.nih.gov/geo/) under accession number GSM2412020. The FASTQ files of bioChIP-seq and ATAC-seq were trimmed with Trim Galore! v0.6.7 and mapped to the mm10 mouse reference genome using Bowtie 2 v2.4.4 with default parameters (Langmead and Salzberg 2012; https://github.com/FelixKrueger/TrimGalore). All alignments were further sorted by Picard Tools v2.27.2 and filtered by SAMtools v1.14 with MAPQ ≥ 10 (Broad Institute) (Li et al. 2009; http://broadinstitute.github.io/picard). The PCR duplicates were removed using Picard Tools v2.27.2. Peaks were identified using MACS2 v2.2.7.1 with a cutoff of FDR < 0.05 and annotated with HOMER v4.11 (Zhang et al. 2008; Heinz et al. 2010). For visualization, RPGC normalized bigWig files were generated using deepTools bamcoverage v3.5.0 (Ramírez et al. 2014).

## Data access

All raw and processed sequencing data generated in this study have been submitted to the NCBI Gene Expression Omnibus (GEO; https://www.ncbi.nlm.nih.gov/geo/) under accession numbers GSE218628 and GSE220724. The codes used in this study are available at GitHub (https://github.com/marcottelab/iTF-seq) and as Supplemental Code.

## Supplementary Material

Supplement 1

Supplement 2

Supplement 3

Supplement 4

Supplement 5

Supplement 6

Supplement 7

Supplement 8

Supplement 9
