# Supplementary material for: Systematic mapping of TF-mediated cell fate changes by a pooled induction coupled with scRNA-seq and multi-omics approaches
Source: Genome Res. 2024 Mar;34(3):484–97. doi: 10.1101/gr.277926.123 (PMC11067882; doi:10.1101/gr.277926.123)
Supplement: Supplement 8 [file Supplelmental_Figure_S1_S8.pdf]

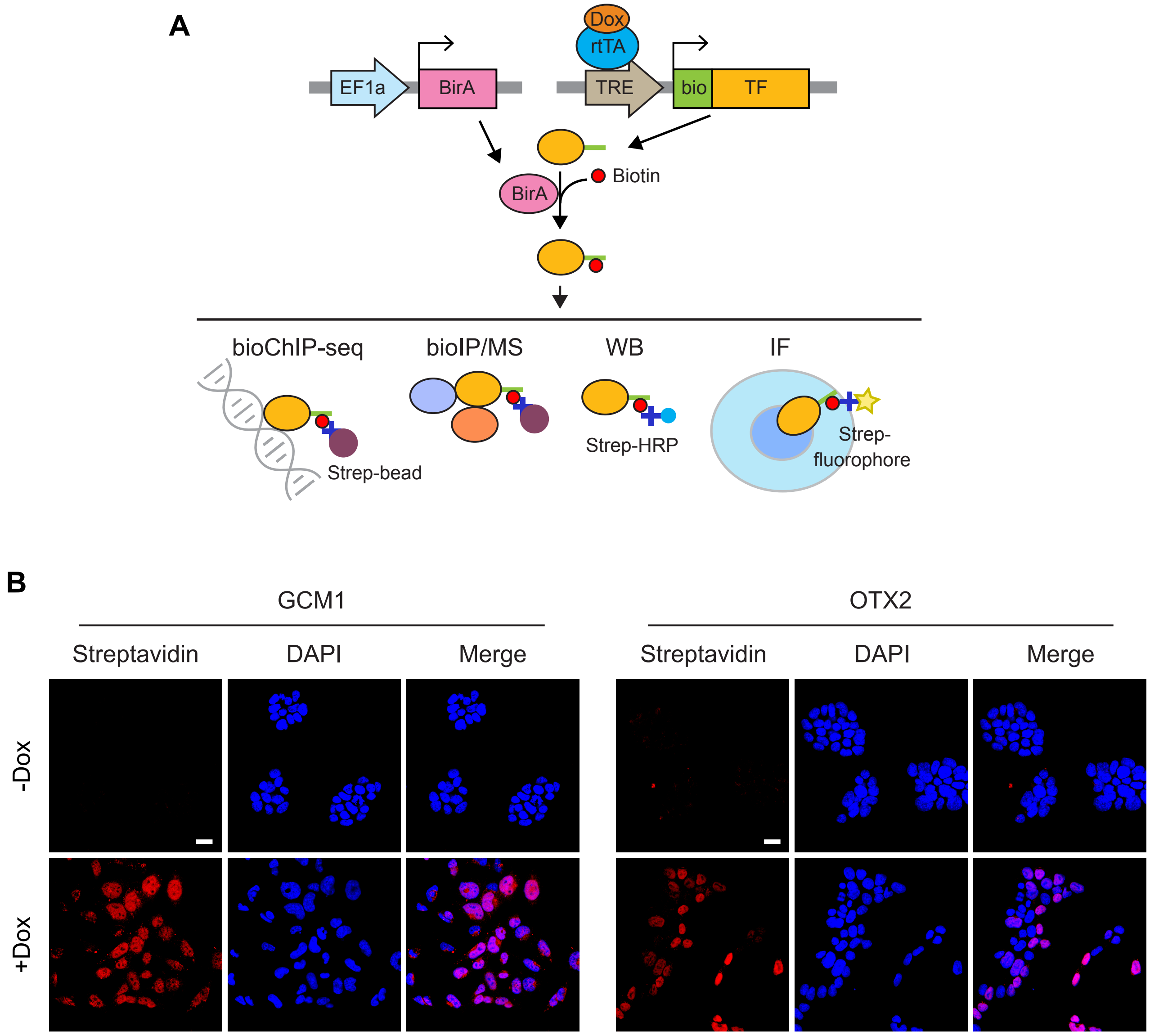

Figure S1. Downstream applications of metabolic biotinylation of TFs
(A) Schematic representation of the SBFB system and its diverse applications.
(B) Visualization of biotinylated Gcm1 and Otx2 induced by treatment of doxycycline (Dox, 0.5 μg/mL) for 2 days. Immunofluorescence was performed using streptavidin-fluorophore (Alexa Fluor™ 555 conjugate, Thermo Fisher Scientific, S21381). Scale bar: 20 μm.

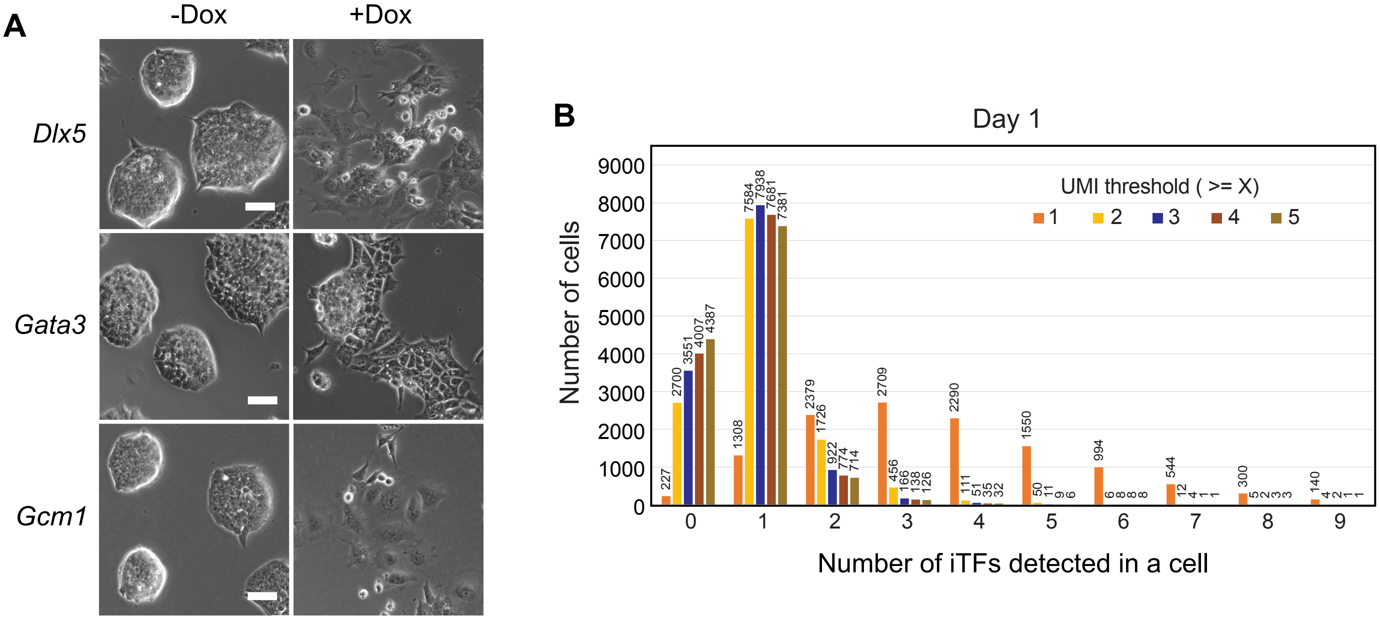

Figure S2. Morphology of representative individual iTF lines upon induction

(A) Morphology of representative individual iTF lines under uninduced condition (-Dox) and upon induction of TFs after treatment with Dox (0.5 µg/mL) for 2 days. Scale bars = 50 µm.

(B) The number of cells after 1 day of induction according to the number of detected iTFs and UMI thresholds. UMI threshold 3 shows the highest number of cells expressing a single iTF (7,938 cells). Cells without detected iTFs under UMI threshold 1 (227 cells) were used as control.

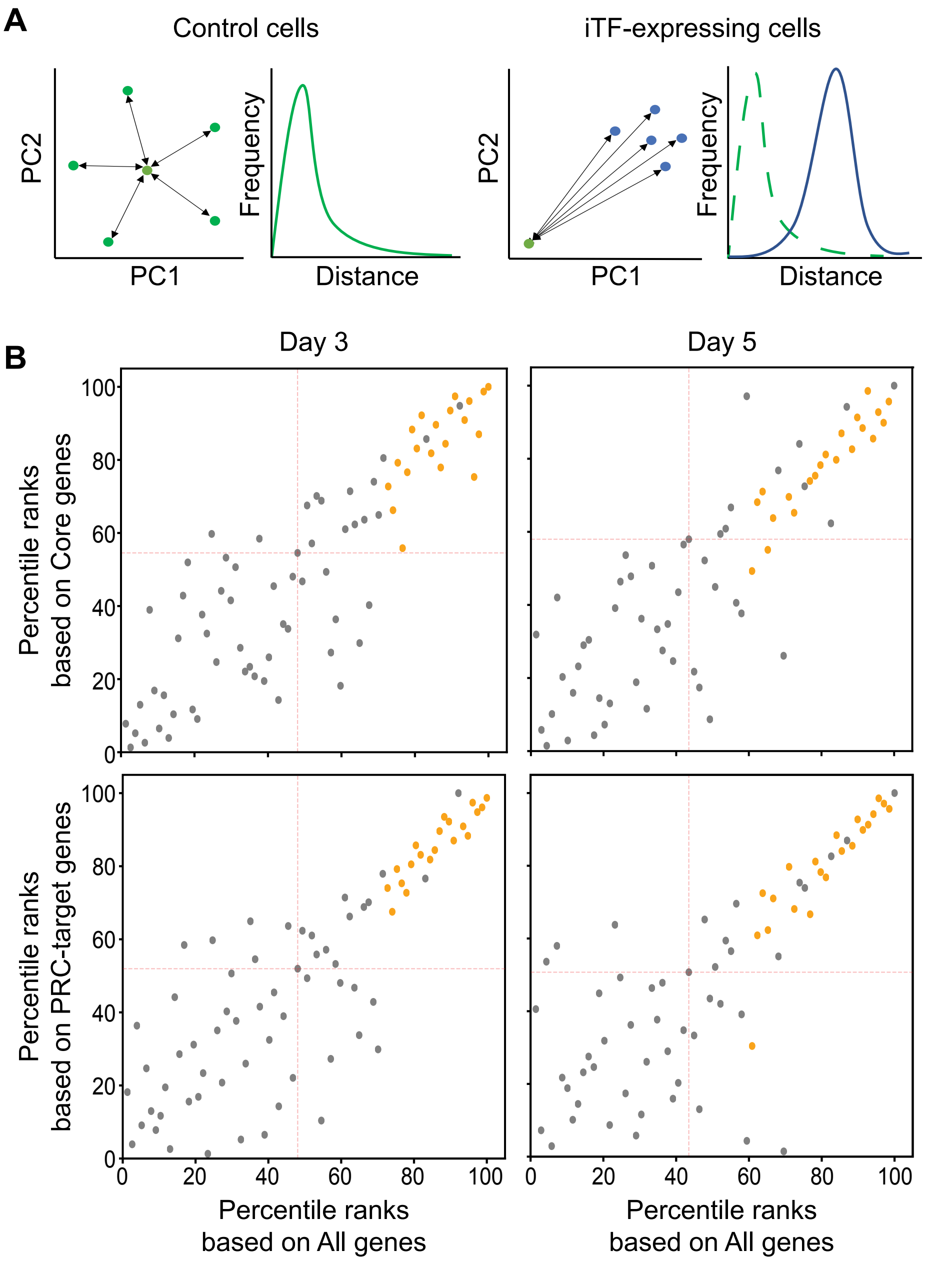

Figure S3. Determining cell fate changes by calculating differentiation index and fate-changing potential of iTFs

(A) Schematic view of differentiation index calculation. Distances between control cells and their centroid were calculated based on principal components (PC), and the distribution was right-skewed (left panels). Then, distances between iTF-overexpressing cells and the centroid of control cells were measured and compared with those of control cells (right panels).

(B) Comparison between differentiation indexes based on All genes (x-axis) and Core genes (y-axis of top plots) or PRC-target genes (y-axis of bottom plots) after 3 and 5 days of induction. Since the differentiation indexes for each gene group have different ranges, percentile ranks were used. TFs with cell fate conversion potential are marked with orange. Red dashed lines show the location of control cells.

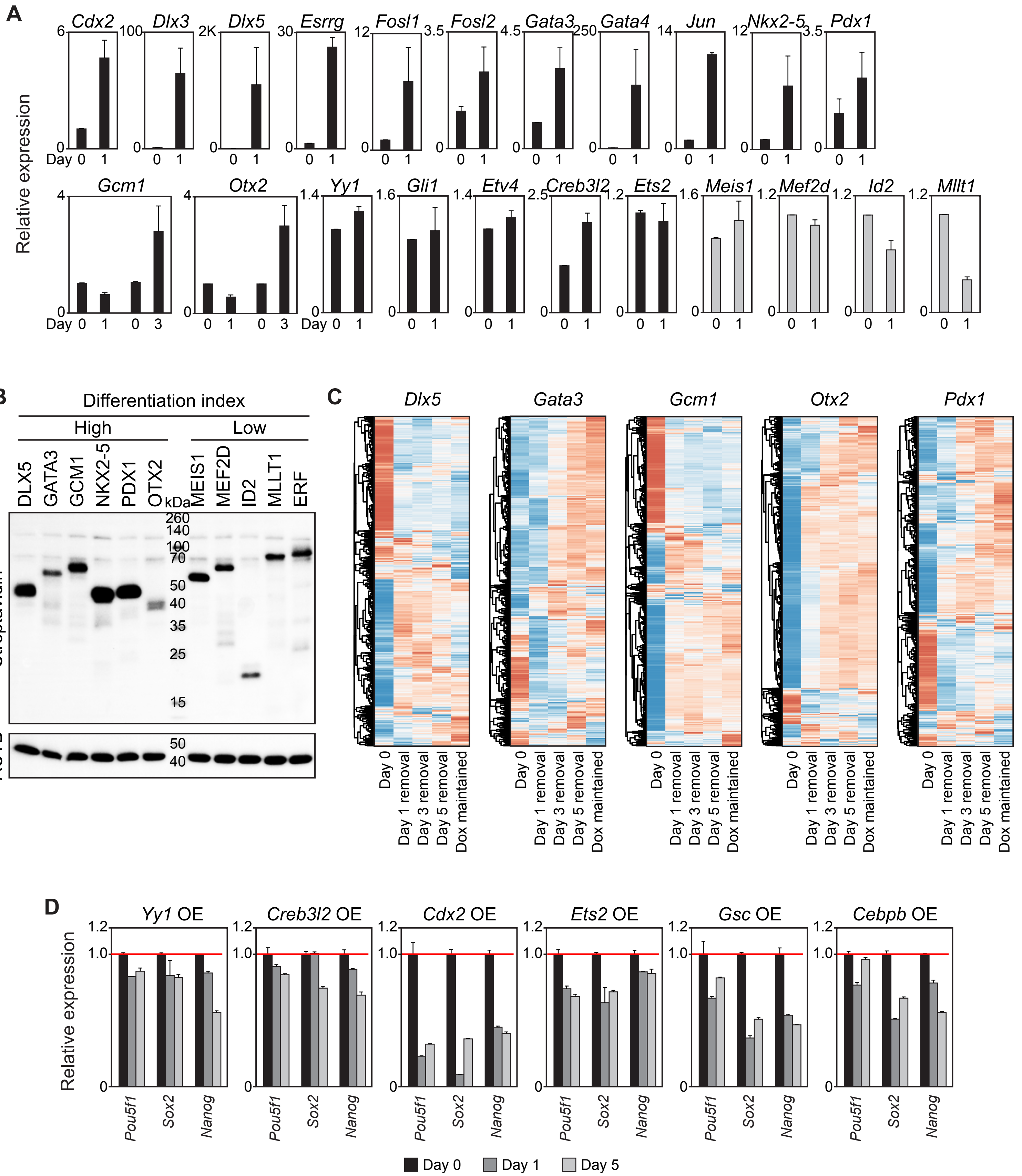

A
Relative expression
Cdx2
Dlx3
Dlx5
Esrrg
Fosl1
Fosl2
Gata3
Gata4
Jun
Nkx2-5
Pdx1
Gcm1
Otx2
Yy1
Gli1
Etv4
Creb3l2
Ets2
Meis1
Mef2d
Id2
Mllt1
Day
B
Differentiation index
High
Low
DLX5
GATA3
GCM1
NKX2-5
PDX1
OTX2
MEIS1
MEF2D
ID2
MLLT1
ERF
kDa
Streptavidin
ACTB
C
Dlx5
Gata3
Gcm1
Otx2
Pdx1
Day 0
Day 1 removal
Day 3 removal
Day 5 removal
Dox maintained
D
Yy1 OE
Creb3l2 OE
Cdx2 OE
Ets2 OE
Gsc OE
Cebpb OE
Relative expression
Pou5f1
Sox2
Nanog
Day 0
Day 1
Day 5

Figure S4. Stable differentiation by induction of potent TFs

(A) Relative expression of the endogenous TF following the overexpression of the ectopic TF with high and low differentiation index. Individual TFs were induced by the addition of doxycycline (Dox, 0.5 μg/ml) for 1 day or 3 days, and the measurements were performed using RT-qPCR. Non-treated cells (Day 0) were used as the control. Error bars indicate mean ± SD (n = 2).

(B) Western blot analysis of expression levels for ectopically expressed TFs by doxycycline treatment (Dox, 0.5 μg/ml) for 2 days. The asterisk (*) denotes non-specific bands.

(C) Heatmaps showing clustering analysis results for genes exhibiting higher expression variance (total 3,000) upon induction of each TF. Cells were treated with doxycycline (Dox, 0.5 μg/ml) for 1, 3, or 5 days, followed by Dox removal, and then maintained for a total of 7 days. Non-treated cells (Day 0) and cells maintained with Dox for 7 days (Dox maintained) were used as controls. The values represent Z-scores (standard scores) of each gene's expressions across conditions.

(D) Relative expression of pluripotency markers, *Pou5f1*, *Sox2*, and *Nanog*, in cells following 1 day or 5 days of induction of indicated ectopic TFs using Dox (0.5 μg/ml), as measured by RT-qPCR. Non-treated cells (Dox 0) were used as the control. Error bars indicate mean ± SD (n = 2).

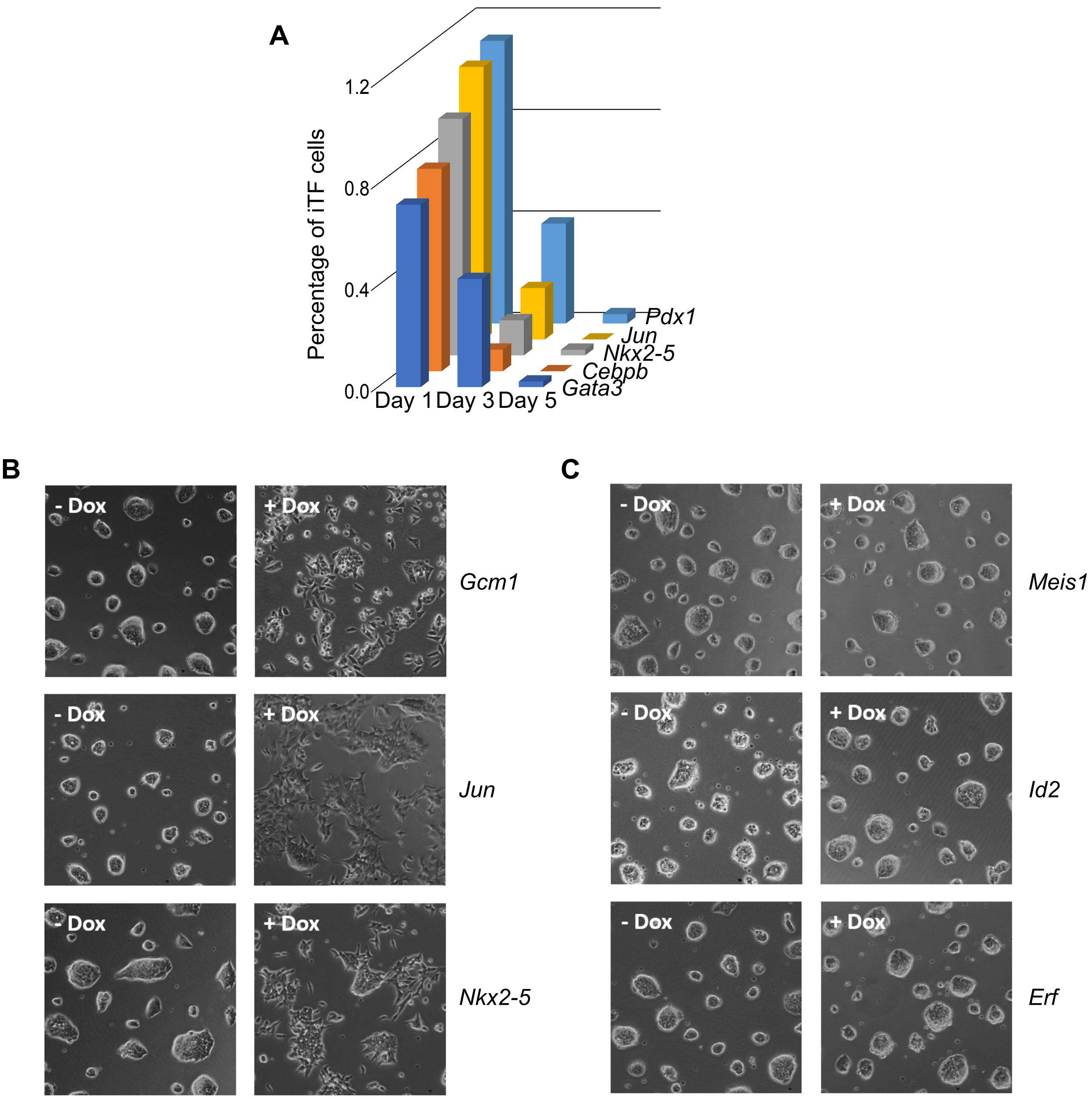

Figure S5. TF induction and cell morphology

(A) Changes in percentage of cell populations expressing indicated TFs among all cells upon induction.

(B) Rapid changes in ES cell morphology upon induction of representative TFs (1 day) with high differentiation index shown in Fig. 3A.

(C) Cell morphology upon induction of TFs with low differentiation index (1 day).

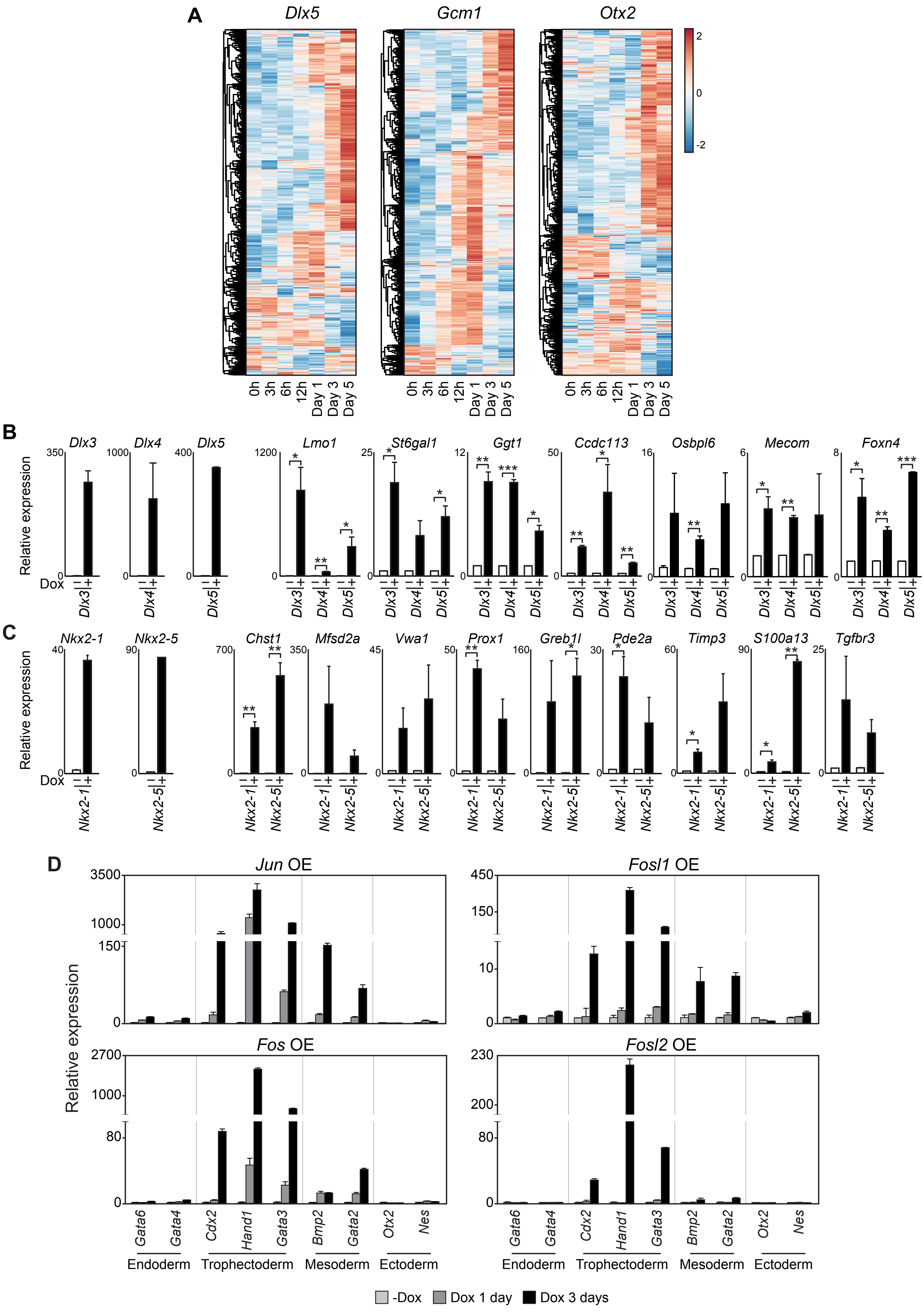
A
Dlx5
Gcm1
Otx2
2
0
-2
0h
3h
6h
12h
Day 1
Day 3
Day 5
B
Relative expression
Dox
Dlx3
Dlx4
Dlx5
Lmo1
St6gal1
Ggt1
Ccdc113
Osbpl6
Mecom
Foxn4
C
Relative expression
Dox
Nkx2-1
Nkx2-5
Chst1
Mfsd2a
Vwa1
Prox1
Greb1l
Pde2a
Timp3
S100a13
Tgfbr3
D
Relative expression
Jun OE
Fosl1 OE
Fos OE
Fosl2 OE
Gata6
Gata4
Cdx2
Hand1
Gata3
Bmp2
Gata2
Otx2
Nes
Endoderm
Trophectoderm
Mesoderm
Ectoderm
-Dox
Dox 1 day
Dox 3 days

Figure S6. Rapid induction of TFs alters transcriptome dynamics and activates lineage marker genes
(A) Heatmaps illustrate the expression profiles of cells overexpressing individual TFs at different time points. Cells were treated with doxycycline (Dox, 0.5 μg/ml) for 3h, 6h, 12h, 1 day, 3 days, or 5 days, and subsequent transcriptome profiling was performed vis RNA-seq. Non-treated cells (0h) were used as the control. The values represent Z-scores (standard scores) of each gene's expressions across conditions.
(B, C) Relative expression levels of lineage markers associated with Dlx3/Dlx4/Dlx5 family TFs (B) and Nkx2-1/Nkx2-5 family TFs (C). Measurements were conducted in cells overexpressing each TF indicated TFs, induced by treating with doxycycline (Dox, 0.5 μg/ml) for 2 days, as measured by RT-qPCR. Non-treated cells (-Dox) were used as the control. Error bars indicate mean ± SD (n = 2). Statistical significance was determined using Student's t-test (*$p < 0.05$, **$p < 0.01$, ***$p < 0.001$).
(D) Relative expression levels of three germ layers and trophectoderm marker genes in cells upon induction of Jun/Fos family TFs. The induction of individual TFs was achieved by treating cells with doxycycline (Dox, 0.5 μg/ml) for 2 days. Non-treated cells (-Dox) were used as the control. Error bars indicate mean ± SD (n = 2).

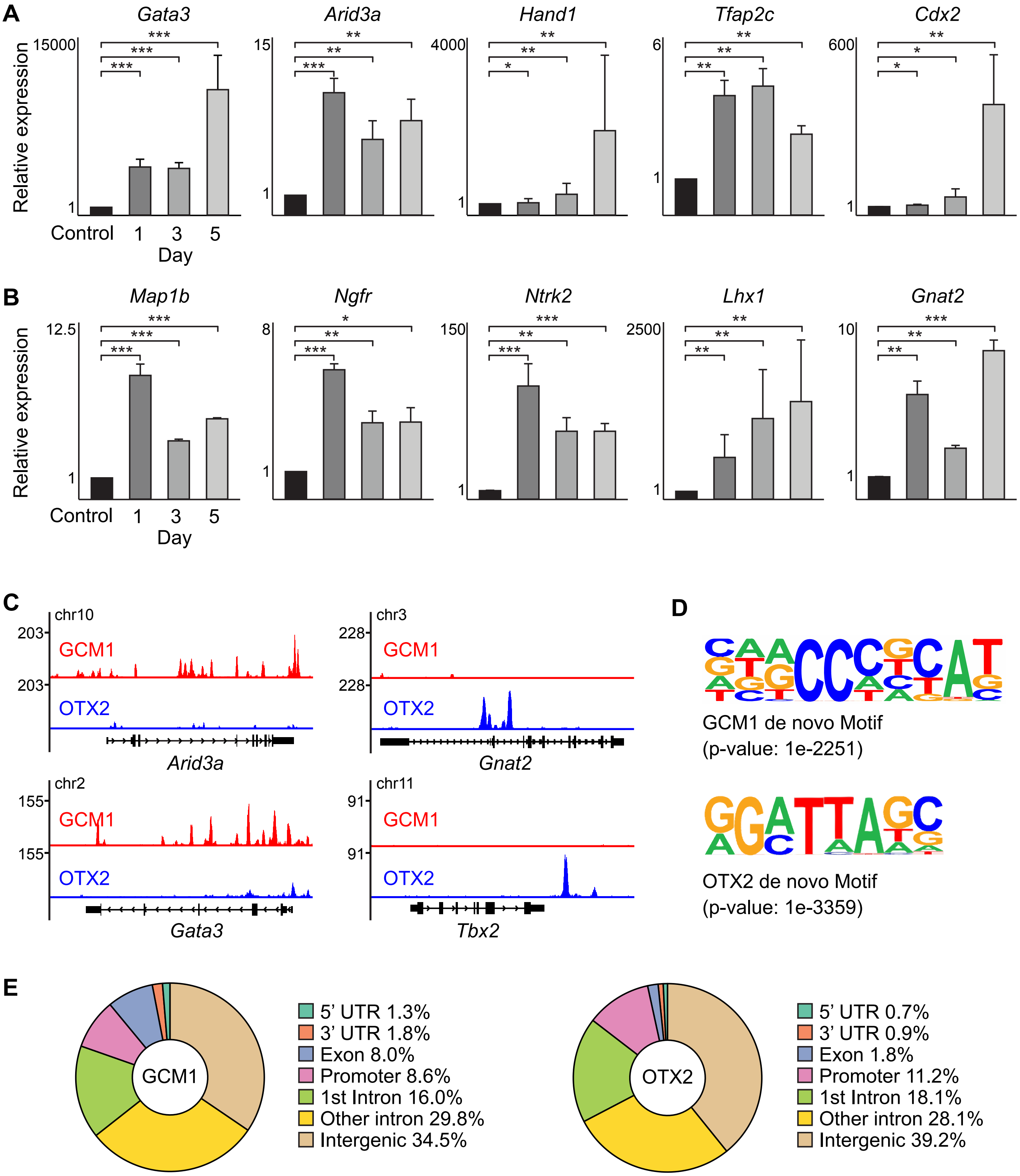
A
Gata3
Arid3a
Hand1
Tfap2c
Cdx2
Relative expression
15000
15
4000
6
600
1
***
**
*
Control
1
3
5
Day
B
Map1b
Ngfr
Ntrk2
Lhx1
Gnat2
12.5
8
150
2500
10
C
chr10
203
GCM1
OTX2
Arid3a
chr3
228
Gnat2
chr2
155
Gata3
chr11
91
Tbx2
D
GCM1 de novo Motif
(p-value: 1e-2251)
OTX2 de novo Motif
(p-value: 1e-3359)
E
GCM1
5' UTR 1.3%
3' UTR 1.8%
Exon 8.0%
Promoter 8.6%
1st Intron 16.0%
Other intron 29.8%
Intergenic 34.5%
OTX2
5' UTR 0.7%
3' UTR 0.9%
Exon 1.8%
Promoter 11.2%
1st Intron 18.1%
Other intron 28.1%
Intergenic 39.2%

Figure S7. Gcm1 and Otx2 medicated cell fate conversion

(A, B) Expressions of placenta and trophoblast marker genes in *Gcm1*-overexpressing cells (A) or neurogenesis-related genes in *Otx2*-overexpressing cells (B) compared to control cells. All data are from three independent samples (SEM: * = 0.01 < P < 0.05, ** = 0.001 < P < 0.01, *** = P ≤ 0.001).

(C) Representative signal track images depicting occupancy of GCM1 or OTX2 around specific genes. (trophoblast lineage; *Arid3a* and *Gata3*, neuronal lineage; *Gnat2* and *Tbx2*).

(D) De novo motifs of GCM1 and OTX2 top peaks identified by HOMER.

(E) Genomic distributions of GCM1 or OTX2 binding peaks.

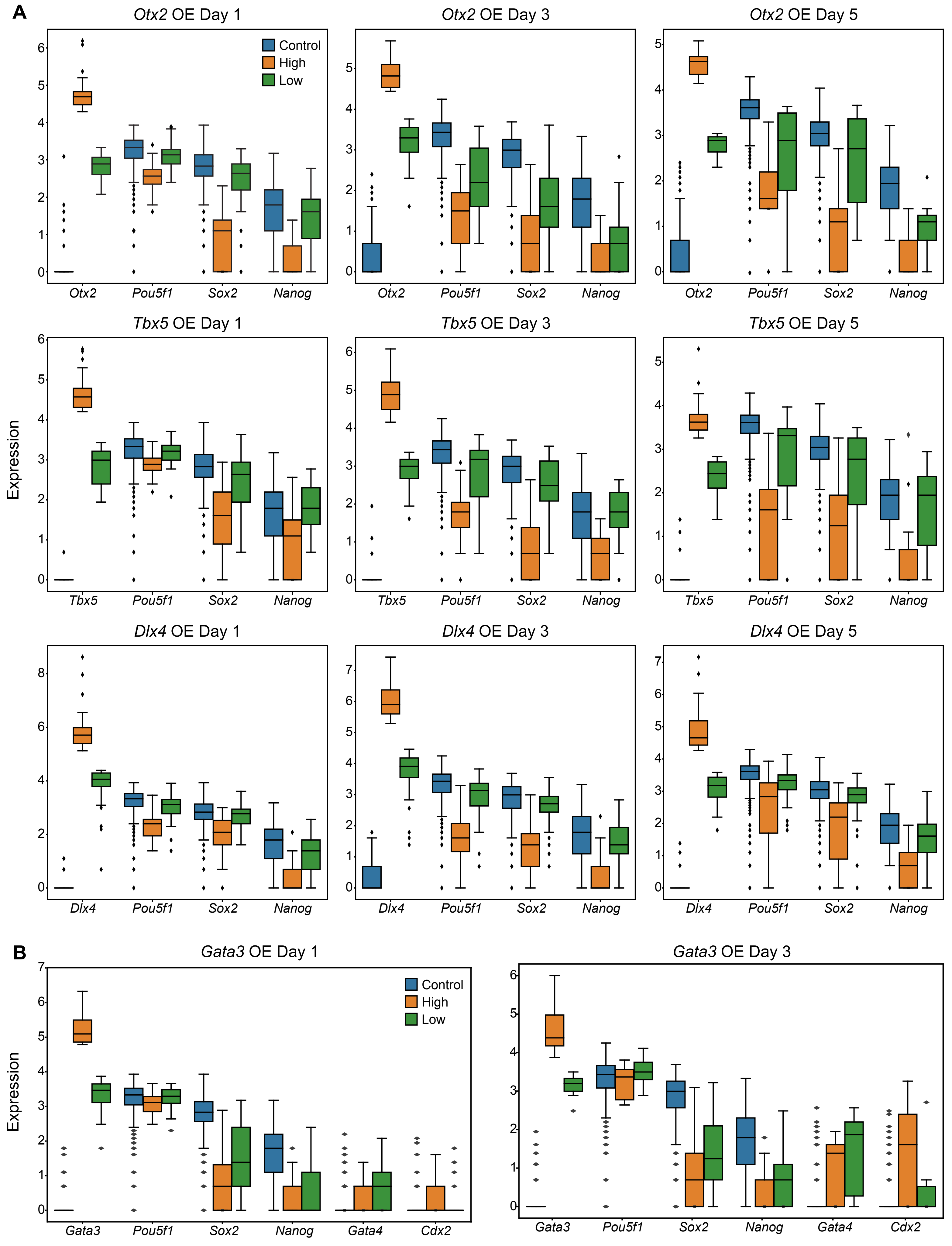
A
Otx2 OE Day 1
Otx2 OE Day 3
Otx2 OE Day 5
Control
High
Low
Otx2
Pou5f1
Sox2
Nanog
Tbx5 OE Day 1
Tbx5 OE Day 3
Tbx5 OE Day 5
Expression
Tbx5
Pou5f1
Sox2
Nanog
Dlx4 OE Day 1
Dlx4 OE Day 3
Dlx4 OE Day 5
Dlx4
Pou5f1
Sox2
Nanog
B
Gata3 OE Day 1
Gata3 OE Day 3
Control
High
Low
Expression
Gata3
Pou5f1
Sox2
Nanog
Gata4
Cdx2

Figure S8. Variation in pluripotency marker expression based on ectopically expressed TF induction levels

(A) Graphs illustrating the expression levels (log-normalized count) of pluripotency markers, *Pou5f1*, *Sox2*, and *Nanog*, in cells with varying levels of ectopically expressed TFs indicated. The induced cells are categorized into two groups: high induction (top 33%) and low induction (bottom 33%). Uninduced control cells were used as the baseline for comparison.

(B) The level of ectopic *Gata3* affects *Gata4* (primitive endoderm marker) vs. *Cdx2* (trophectoderm marker) expression.
